# Technic of Secondary Suture

**Published:** 1919

**Authors:** 


***Technic of Secondary Suture.*** By Dr. EHRENPREIS. *La Presse Médicale*, September 23, 1918.

Secondary suture is resorted to in war wounds when primary suture proves impossible, either for military reasons, or because of the condition of the wounds. Even then, delayed primary sutures can sometimes be practiced, one surgeon removing the projectile and excising its track, and another surgeon suturing the wound later on. Furthermore the author considers that primary suture can be performed without fear a few days after receipt of the wound, as long as this proves strictly " non-infected ".

When primary suture cannot be practiced, the wound is given some antiseptic treatment (with Dakin's solution, or magnesium chloride, or simply dry dressing, and heliotherapy) and then sutured as soon as possible.

Secondary suture requires the following conditions :

(1) The wound must be sterile. Bacteriological control should be resorted to and should give repeated negative results. The secretions to be tested should be taken from the " culs-de-sac " and corners of the wound, where the micro-organisms mostly develop. Simple smears do not prove sufficient, but cultures should be tried especially for the research of streptococci. If the wound appears uniformly red, covered with small granulations; if it does not bleed easily, and does not show any trace of lymphangitis in the neighborhood; if no pain, no fever, no tension of the limb are present, the conditions of the wound allow of its being sutured.

(2) The wound should be as even as possible. No "dead space" should be allowed to remain, as its cavity would prove most favorable for the further development of infection. If such spaces exist, the tissues should be brought together during the operation so as to fill them up exactly. If some severe fracture has taken place, the surgeon must postpone the suture until the cavity in the bone is completely filled up. In doing so he should avoid suturing the tissues over some neo-pseudarthrosis, or some sequestra which might later on be the cause of fistula. Likewise a suppurating pleural cavity should not be closed until the lungs and pleura can be brought into contact by any process, after having been given the Carrel treatment.

(3) The closure of the wound should be obtained without any tension of the tissues. Even if a great loss of substance has occurred and no autoplastic proceedings allow the repairing of this, it is necessary to wait until the activity of the injured tissues has occasioned some sufficient repair. Sometimes, however, the edges of the wound appear intensely retracted, but this may not be due to real loss of substance. It happens often after treatments necessitating some irrigating process. But the appliance of dry, or better still of fat, dressings for a few days before the operation easily removes that appearance,

*Technic of the Secondary Suture.* It is advisable to clean most carefully the wound and all surrounding parts on the day before operating. This may be done by scrubbing with soap, swabbing with ether, and leaving a dressing soaked with some alcoholic solution. General anesthesia proves most convenient. When the patient is anesthetized, a second cleansing with soap and ether should be given.

*Incision.* This is done about 2 mm. away from the edges of the wound, in sound tissues, and should penetrate deeper than the subcutaneous tissue. This should be cut off in one piece together with the dermis and epidermis.

*Resection.* The wound must be excised as a tumor would be. All the buds, nodular and fibrous tissues must be removed most carefully from the remotest corners of the wound.

*Repairing of the Lesions.* All necessary suture of muscles, tendons and nerves is done after all clots of blood and small hanging pieces of muscles or aponeurosis have been carefully removed. If necessary, muscles should be freed from all abnormal adhesions. Complete hemostasis having been secured, the whole wound is irrigated with ether before suturing.

*Suture.* This consists of as complete a restoration of all anatomical layers as possible. The suturing points of muscles and tendons should not be very tight. Fine catgut should be used, and knotted loosely.

The suture of the skin must cover up the whole operatory surface and autoplastic grafts should be used if necessary.

Drainage is useful only in very severe wounds with important losses of substance. In such cases it is wise to institute some filiform drainage for the first 24 or 48 hours.

If post-operatory infection is feared, the skin should only be sutured at the extremities of the wound; in the center, loose sutures should be put in and tightened only after 2 or 3 days.

All threads can usually be taken off after 10 or 12 days.

